# Structural Characterization of Humanized Nanobodies with Neutralizing Activity against the *Bordetella pertussis* CyaA-Hemolysin: Implications for a Potential Epitope of Toxin-Protective Antigen

**DOI:** 10.3390/toxins8040099

**Published:** 2016-04-01

**Authors:** Aijaz Ahmad Malik, Chompounoot Imtong, Nitat Sookrung, Gerd Katzenmeier, Wanpen Chaicumpa, Chanan Angsuthanasombat

**Affiliations:** 1 Graduate Program in Immunology, Department of Immunology, Faculty of Medicine Siriraj Hospital, Mahidol University, Bangkok 10700, Thailand; ajaz_me@hotmail.com; 2 Bacterial Protein Toxin Research Cluster, Institute of Molecular Biosciences, Mahidol University, Salaya Campus, Nakornpathom 73170, Thailand; chompou_ron@hotmail.com (C.I.); katzenmeier.ger@mahidol.ac.th (G.K.); 3 Department of Parasitology and Center of Excellence on Therapeutic Proteins and Antibody Engineering, Faculty of Medicine Siriraj Hospital, Mahidol University, Bangkok 10700, Thailand; 4 Department of Research and Development, Faculty of Medicine Siriraj Hospital, Mahidol University, Bangkok 10700, Thailand; nitat.soo@mahidol.ac.th; 5 Laboratory of Molecular Biophysics and Structural Biochemistry, Biophysics Institute for Research and Development (BIRD), Bangkok 10160, Thailand

**Keywords:** *Bordetella pertussis*, CyaA-hemolysin, CyaA-RTX, intermolecular docking, VH/V_H_H, phage display

## Abstract

Previously, the 126-kDa CyaA-hemolysin (CyaA-Hly) fragment cloned from *Bordetella pertussis*—the causative agent of whooping cough—and functionally expressed in *Escherichia coli* was revealed as a key determinant for CyaA-mediated hemolysis against target erythrocytes. Here, phagemid-transfected *E. coli* clones producing nanobodies capable of binding to CyaA-Hly were selected from a humanized-camel VH/V_H_H phage-display library. Subsequently verified for binding activities by indirect ELISA and Western blotting, four CyaA-Hly-specific nanobodies were obtained and designated according to the presence/absence of V_H_H-hallmark amino acids as V_H_H2, VH5, VH18 and V_H_H37. *In vitro* neutralization assay revealed that all four ~17-kDa His-tagged VH/V_H_H nanobodies, in particular V_H_H37, which were over-expressed as inclusions and successfully unfolded-refolded, were able to effectively inhibit CyaA-Hly-mediated hemolysis. Phage-mimotope searching revealed that only peptides with sequence homologous to Linker 1 connecting Blocks I and II within the CyaA-RTX subdomain were able to bind to these four CyaA-Hly-specific nanobodies. Structural analysis of V_H_H37 via homology modeling and intermolecular docking confirmed that this humanized nanobody directly interacts with CyaA-RTX/Linker 1 through multiple hydrogen and ionic bonds. Altogether, our present data demonstrate that CyaA-RTX/Linker 1 could serve as a potential epitope of CyaA-protective antigen that may be useful for development of peptide-based pertussis vaccines. Additionally, such toxin-specific nanobodies have a potential for test-driven development of a ready-to-use therapeutic in passive immunization for mitigation of disease severity.

## 1. Introduction

Pertussis or whooping cough is a highly contagious respiratory disease of humans caused by an aerobic, non-spore-forming, Gram-negative coccobacillus, *Bordetella pertussis* [1]. In recent years, there has been an upsurge of whooping cough among elderly people [1] whose vaccination-induced protective immunity waned-off due to the lack of natural boosters caused by a decrease of circulating pathogens as a result of mass vaccination [2]. This pertussis-causative pathogen secretes several virulence factors among which is the adenylate cyclase-hemolysin toxin (CyaA) that plays an important role during the early phase of infection [3,4].

CyaA is a 1706-residue long bi-functional protein which consists of an N-terminal adenylate cyclase (AC) catalytic domain (residues 1–400) and a C-terminal pore-forming or hemolysin (Hly) domain (residues 401–1706) [4]. Upon entry into the host cells, catalytic function of the AC domain is activated by endogenous calmodulin, leading to supra-physiological levels of cAMP that would result in cell death and disruption of the host innate immune responses [5,6]. The CyaA-Hly domain which contains a hydrophobic pore-forming subdomain (residues 500–700) has the ability to form cation-selective channels causing lysis of target cells [7,8]. There is also an RTX (Repeat-in-ToXin) subdomain (residues 1006–1613) which harbors ~40 repeats of Gly-Asp-rich nonapeptides [9] and is organized into five structurally similar blocks (Blocks I-V) connected by linker sequences (Linkers 1–4) of variable lengths [10,11]. CyaA is stabilized by extracellular Ca^2+^ ions which serve as a structure-stabilizing bridge in a β-roll structure within each RTX-Block region [10,11,12]. Moreover, CyaA is synthesized as an inactive precursor which requires a palmitoyl group be added at Lys^983^ by CyaC acyltransferase [7,13,14].

The CyaA-RTX subdomain is involved in toxin binding to target cells through the α_M_β_2_-integrin receptor (also known as CD11b/CD18) expressed on the surface of cells in the myeloid lineage, e.g., neutrophils and macrophages [15]. CyaA also exerts its hemolytic activity against sheep erythrocytes, although they lack the α_M_β_2_-intergrin receptor, suggesting the possibility of an alternative pathway for target cell recognition via the RTX subdomain [8,11]. In addition, we have shown that the 126-kDa truncated CyaA-Hly fragment still retains high hemolytic activity independent of the *N*-terminal AC domain [8,16].

In our recent studies, we have identified the involvement of Linker 2 of the CyaA-RTX subdomain in binding with sheep erythrocytes [11]. We have also successfully generated specific VH/V_H_H nanobodies against many targets, including viral proteins, snake venoms and botulinum neurotoxin, from an established humanized VH/V_H_H phage-display library [17,18,19,20]. In the present study, CyaA-Hly-specific humanized VH/V_H_H nanobodies were obtained and their characteristics of hemolysis inhibition on target erythrocytes were revealed, suggesting a possible role of such humanized nanobodies as a novel adjunctive anti-pertussis agent. Moreover, we have identified the region on Linker 1 connecting Blocks I and II within the CyaA-RTX subdomain that could be a potential neutralizing epitope of CyaA-protective antigen.

## 2. Results and Discussion

### 2.1. Isolated CyaA-Hly-Specific Nanobodies with Different CDR-3 Loops

Previously, we have succeeded in producing phage-display nanobodies, *i.e.*, human ScFvs and humanized-camel VHs/V_H_Hs, which can bind specifically to functional regions of different target proteins, e.g., influenza A virus, hepatitis C viral proteins, *Naja kaouthia* phospholipase-A2 and botulinum neurotoxin-type A [17,18,19,20,21]. Here, attempts were made to generate CyaA-Hly-specific nanobodies from a humanized-camel VH/V_H_H phage-display library. After single-round bio-panning against CyaA-Hly, a total of forty phage-transformed *E. coli* clones were selected and subjected to PCR analysis for initial verification of the presence of VH/V_H_H-coding sequences. Among these selected clones, thirty-four clones were *vh*/*v_h_h*-positive as they yielded 600-bp amplicons indicative of recombinant *vh*/*v_h_h*-inserted phagemids (see Figure S1a). As subsequently revealed by Western blotting, all *vh*/*v_h_h*-positive clones were able to express corresponding soluble VH/V_H_H proteins (~17–22 kDa) which were immuno-reactive to anti-E tag antibodies (see Figure S1b), indicating the presence of a such epitope tag which was incorporated in the *C*-terminus of target VH/V_H_H proteins. Hence, our established humanized phage library likely contains high percentage of phages (*i.e.*, ~85%) harboring VH/V_H_H*-*expressing inserts.

**Figure 1:**
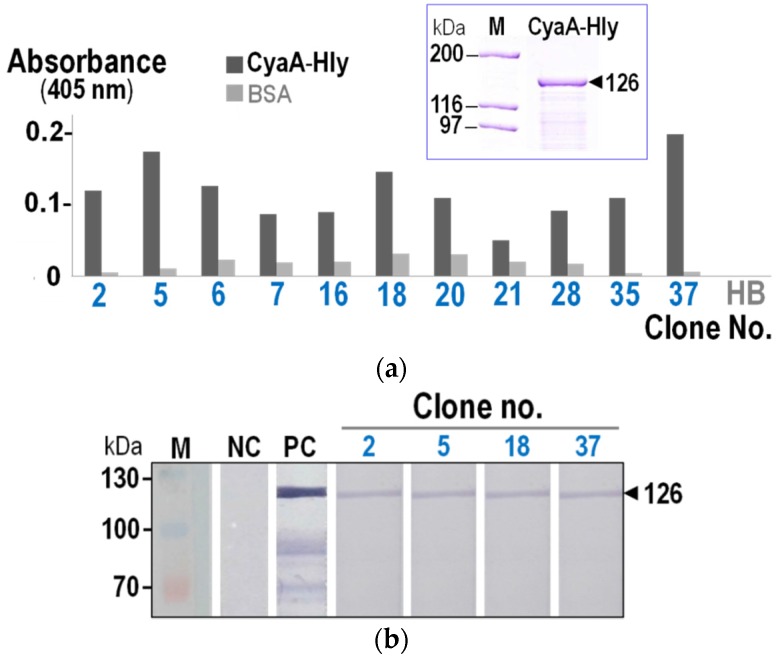
(**a**) Indirect ELISA results of lysates from selected *E. coli* clones expressing VHs/V_H_Hs that give OD_405_ signals to the immobilized CyaA-hemolysin (CyaA-Hly) toxin (■) at least twice above the BSA control (■). Normal HB2151-*E. coli* lysate (HB) was used as a background control. *Insert,* SDS-PAGE analysis (Coomassie brilliant blue-stained 10% gel) of purified CyaA-Hly used in the assay. M, protein-molecular mass standards. (**b**) Western blotting results showing specific binding of VHs/V_H_Hs of four clones (*nos.* 2, 5, 18 and 37) to CyaA-Hly. M, prestained protein standards. PC, SDS-PAGE-separated CyaA-Hly probed with rabbit anti-RTX polyclonal antisera. NC, SDS-PAGE-separated CyaA-Hly probed with normal HB2151-*E. coli* lysate. An arrow indicates the band corresponding to the 126-kDa CyaA-Hly protein.

Due to low-binding specificity of the single-round bio-panning, the VH/V_H_H proteins expressed in the phage-transformed *E. coli* were therefore verified for their binding capability to CyaA-Hly via indirect ELISA and Western blotting. As shown in Figure 1a, lysates from eleven *E. coli* clones (~40%) containing VH/V_H_H proteins gave significant OD_405_ signals to the immobilized CyaA-Hly toxin above the BSA control, reflecting their high-binding activity against the target toxin. Nevertheless, subsequent analysis via Western blotting revealed that only lysates from four of these ELISA-positive clones could give rise to an intense binding signal to SDS-PAGE-separated CyaA-Hly seen as 126-kDa immuno-reactive bands (Figure 1b). The results suggest that these four CyaA-Hly-specific nanobodies were able to recognize a sequential epitope of the denatured target protein whereas the remaining ELISA-positive nanobodies apparently recognized conformation-dependent epitopes that were abolished by SDS denaturation.

**Figure 2:**
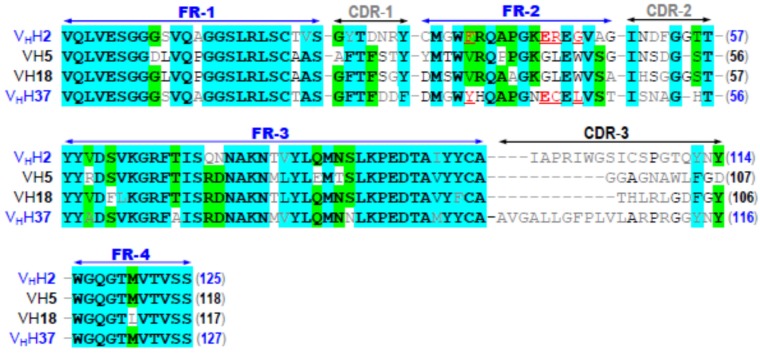
Multiple sequence alignments of the deduced amino acid sequences among the four cloned CyaA-Hly-specific nanobodies, showing their FRs and CDRs. FR-2/V_H_H-hallmark residues found in clone nos. 2 and 37 are underlined and denoted in red. Amino acids are bolded and shaded cyan and green to denote degrees of identity (4/4) and (3/4), respectively.

Multiple alignments of deduced amino acid sequences of the four CyaA-Hly-specific nanobodies for determining CDRs and FRs revealed that their CDR regions which are widely assumed to be responsible for antigen recognition attain a relatively low sequence identity, particularly in the CDR-3 loop (Figure 2), thus implying that these four individual VH/V_H_H nanobodies in parts interact with different regions of such a linear epitope on the CyaA-Hly toxin. Further sequence analysis (Figure 2) revealed that the FR-2 sequences of two clones (designated V_H_H2 and V_H_H37) bear a tetrad amino acid hallmark, *i.e.*, Phe/Tyr^42^_Glu^49^_Arg/Cys^50^_Gly/Phe^52^, which is a signature of variable heavy chain domains, V_H_Hs [22]. In addition, the remaining two clones (designated VH5 and VH18) display the FR-2 feature of a tetrad conventional VH of mammals including human, *i.e.*, Val^42^_Gly^49^_Leu^50^_Trp^52^. A marked difference between V_H_Hs and human VHs found at FR-2/tetrad residues could determine their dissimilarity in hydrophobicity at the variable light chain-binding site as suggested earlier [22]. However, this hallmark has nothing to do with the antigenic specificity of the antibodies since FR-2 is not thought to participate in antigen recognition [22].

### 2.2. In vitro Neutralizing Activity of CyaA-Hly-Specific Nanobodies

**Figure 3:**
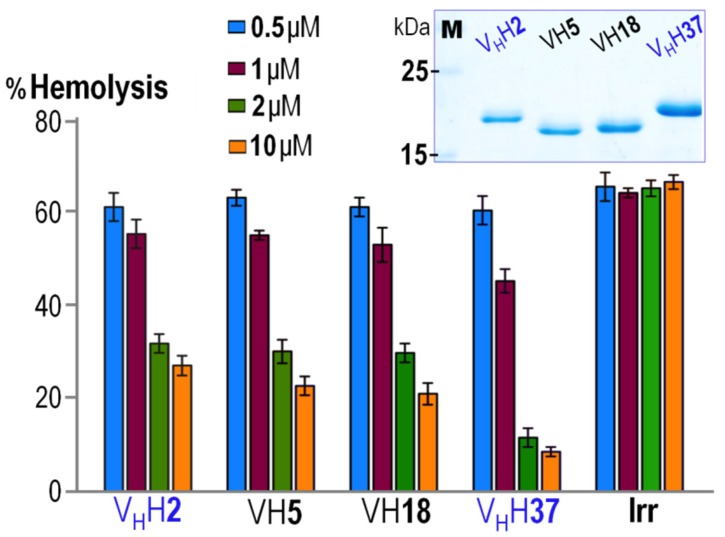
Dose-dependent inhibition of CyaA-Hly-mediated hemolysis by individual CyaA-Hly-specific nanobodies. Purified CyaA-Hly (~10 nM) was pre-incubated with various concentrations (*i.e.*, 0.5, 1, 2 and 10 µM, as denoted by different colors) of purified V_H_H2, VH5, VH18, V_H_H37 and an irrelevant control nanobody (Irr) prior to incubating with sheep erythrocytes in the assay reaction. The extent of inhibition was calculated by percent of hemolysis induced by 0.1% Triton X-100. Error bars indicate standard deviation from assays tested for each sample in triplicate. *Inset*, SDS-PAGE analysis (Coomassie brilliant blue-stained 14% gel) of the purified and refolded nanobodies of ~17 kDa as indicated in the assay. M, protein-molecular mass standards.

Since expression levels of the CyaA-Hly-specific nanobodies obtained in the current system via the *lac* operon promoter were relatively low, a large quantity of their purified soluble forms could not be obtained via anti-E tag affinity chromatography. We thus constructed recombinant plasmids that placed the nanobody genes under control of T7 RNA polymerase-driven system to over-express the individual nanobodies fused at the *C*-terminus with a 6× His tag. Upon IPTG induction, all four nanobodies (~17–20 KDa) were strongly produced as inclusion bodies which were then verified for the presence of a His-affinity tag via Western blotting (see Figure S2) and completely solubilized in phosphate buffer (pH 7.0) supplemented with 8 M urea. The unfolded His-tagged nanobodies were refolded in a Ni^2+^-NTA affinity column via gradients of decreasing urea concentrations and finally a high-yield protein band with >95% purity of each re-natured VH/V_H_H was obtained in urea-imidazole-free phosphate buffer as analyzed by SDS-PAGE (Figure 3, *inset*). Moreover, these refolded nanobodies were able to retain their binding affinity to the immobilized CyaA-Hly toxin via indirect ELISA, suggestive of their native-like folded conformation.

Recently, we have demonstrated that anti-CyaA-RTX antisera can effectively inhibit hemolytic activity of CyaA-Hly against sheep erythrocytes, suggesting that anti-RTX antisera block the capability of CyaA-Hly to bind such target membranes and hence interfere with toxin-mediated hemolysis [11]. Herein, the purified CyaA-Hly-specific nanobodies were further assessed for their ability to inhibit hemolytic activity of the toxin. Toxin neutralization assays were performed by pre-mixing the CyaA-Hly toxin (~10 nM) with varied concentrations of individual nanobodies prior to incubation with target erythrocytes. While CyaA-Hly pre-incubated with an irrelevant nanobody (*i.e.*, V_H_H nanobody selected against the hepatitis C viral NS3/4A protease [17]) retained high hemolytic activity against sheep erythrocytes, a dose-dependent inhibition of CyaA-Hly-induced hemolysis was observed for all individual CyaA-Hly-specific nanobodies (Figure 3). Although all four VH/V_H_H nanobodies at 0.5 or 1 µM concentrations showed negligible effects on hemolysis inhibition, their inhibitory effects were clearly observed at the concentration of 2 µM, implying that the available neutralizing epitopes on the toxin would be sufficiently directed by individual nanobodies with concentrations 200-fold higher than the target toxin, and thus showing their significant inhibition on CyaA-Hly-mediated hemolysis. It is noteworthy that among all VH/V_H_H nanobodies tested for hemolysis inhibition, V_H_H37 is the most effective toxin-neutralizing nanobody. Although both V_H_H37 and V_H_H2 have the characteristic tetrad amino acids in FR-2, higher neutralizing activity of the V_H_H37 nanobody is likely contributed to its CDR-3 loop region whose sequence and length are obviously different from those of the remaining nanobodies (see Figure 2). Altogether, these data suggest that all the purified-refolded nanobodies maintain their native-folded conformation and ability to block CyaA-Hly binding to its target molecule on erythrocyte membranes, thereby neutralizing CyaA-Hly-induced hemolysis. Despite inhibitory capability of the obtained CyaA-Hly-specific nanobodies, nonetheless, no plausible binding site for CyaA-Hly on the erythrocyte membrane has yet been identified. Recently, we have validated the CyaA-Hly binding on sheep erythrocytes by demonstrating that its binding appears as focal associations [11].

### 2.3. CyaA-RTX/Linker 1 Serving as a Potential Epitope for Toxin-Neutralizing Nanobodies

**Figure 4:**
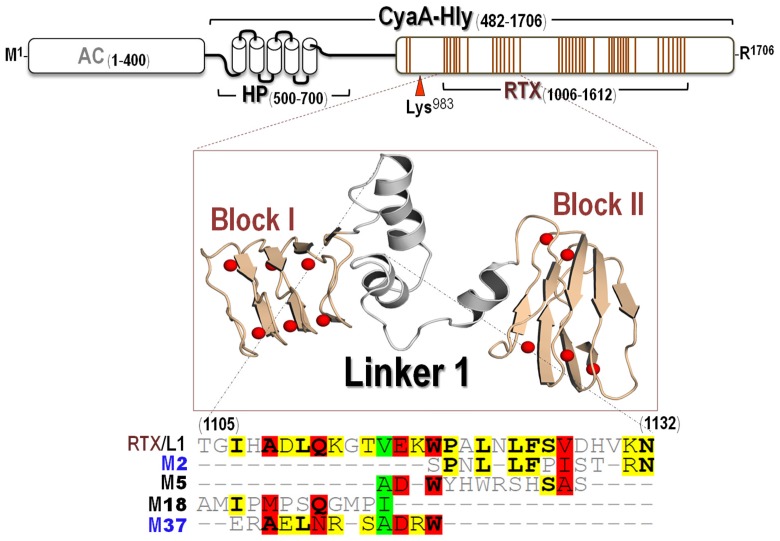
Schematic diagram of CyaA showing adenylate cyclase (AC) and hemolysin (Hly) domains. Five putative helices in the HP region (residues 500–700) are represented by blocks. Palmitoylation site is indicated by Lys^983^. Ca^2+^-binding regions in the RTX subdomain are denoted by multiple lines, each of which corresponds to a single-nonapeptide repeat (Gly-Gly-X-Gly-X-Asp-X-Leu-X). 3D-model of the first two RTX blocks (Blocks I and II) with Linker 1 is shown. Red balls represent Ca^2+^ ions. Multiple sequence alignments of the deduced amino acid sequences of four phage-mimotope peptides (M2, M5, M18 and M37) with sequence of CyaA-RTX/Linker 1 (RTX/L1) are presented. Amino acids are bolded to denote their identity. Degree of conservation among the sequences is highlighted by shading residues with green, red and yellow for 80%, 60% and 40% homology, respectively.

To understand neutralizing mechanisms of these CyaA-Hly-specific nanobodies, it is important to know how they interact with a specific target region on their toxin counterpart. Further attempts were therefore made via phage-mimotope searching to identify a potential epitope region for each specific VH/V_H_H by determining a phage peptide that can bind explicitly to such nanobodies. Four phage clones displaying 12-residue peptides capable of binding to each individual nanobody (*i.e.*, V_H_H2, VH5, VH18 and V_H_H37) were successfully selected and designated mimotopes: M2 (SPNLLFPISTRN), M5 (ADWYHWRSHSSS), M18 (AAMIPMPSQGMP) and M37 (ERAELNRSADRW), respectively (Figure 4).

As described earlier, the CyaA-RTX subdomain (residues 1006–1613) can be organized into five structurally similar blocks, Block I_1080–1138_, Block II_1087–1137_, Block III_1212–1259_, Block IV_1377–1485_, and Block V_1529–1591_, joined by linker sequences (Linkers 1–4) of variable lengths (23 to 49 residues) [9,10,11]. Herein, when the obtained mimotope sequences were multiply aligned with the CyaA-Hly sequence, all these mimotopes were found to match the Linker 1 loop sequence (Thr^1105^ to Asn^1132^) connecting Blocks I and II (Figure 4), thus suggesting that such the RTX-Linker 1 region is a potential neutralizing epitope for these CyaA-Hly-specific nanobodies.

To gain more insights into molecular interactions between individual CyaA-Hly-specific nanobodies and their potential neutralizing epitope (the RTX-Linker 1 region), *in silico* intermolecular docking between two interacting counterparts was performed. Since there is no crystal structure available for CyaA-Hly or its RTX subdomain, a plausible 3D-modeled structure of the CyaA-RTX segment encompassing Block I-Linker1-Block II (CyaA-RTX/BI-II, residues 1006–1210) was constructed based on the known structure of *Pseudomonas* sp. MIS38 lipase (PDB ID: 2ZJ6). Ramachandran plots of backbone-dihedral angles φ against ψ of amino acids in the CyaA-RTX/BI-II modeled structure revealed that over 93% of the total residues are in the allowed conformational region. Thus, this 3D-model is likely to be stereo-chemically sound with a reasonable distribution of torsion angles in the built structure. As can be inferred from Figure 4, the modeled structure of the CyaA-RTX/BI-II region appears to adopt a characteristic of parallel β-roll structures in Blocks I and II connected together by three-helix structure of Linker 1. 3D-modeled structures of four individual VH/V_H_H nanobodies were also constructed using best-fit known-structure templates with a maximum identity including *Acanthamoeba castellanii* profilin II (PDB ID: 1F2K) for V_H_H2, camelid Fab fragment (PDB ID: 4O9H) for VH5, scFv-IL-1B complex (PDB ID: 2KH2) for VH18 and llama V_H_H nanobody (PDB ID: 4HEP) for V_H_H37 with 76%, 78%, 82% and 65% identity, respectively. Moreover, their individual φ/ψ plots indicate that each modeled structure stays in sterically favorable main-chain conformations.

**Figure 5:**
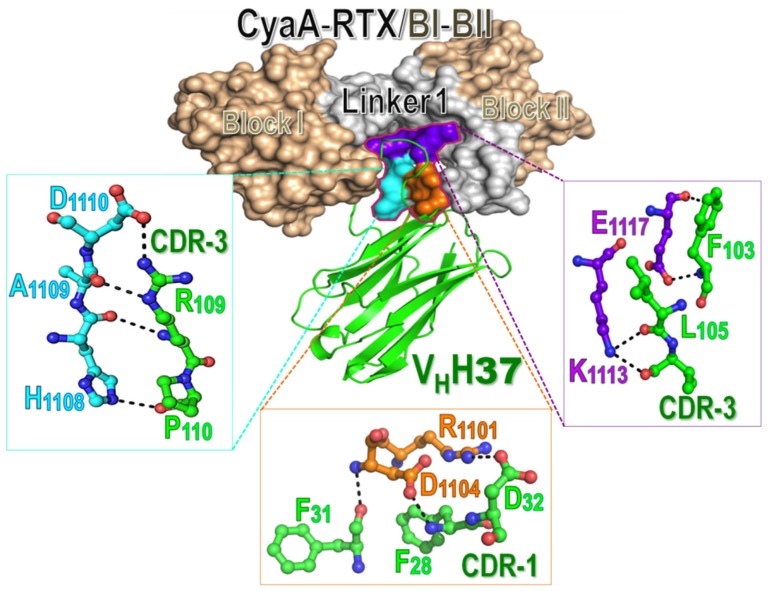
Molecular interactions between the CyaA-RTX/BI-II segment (surface representation, Blocks I and II colored in wheat and Linker 1 in gray) and V_H_H37 (green schematic ribbon) which illustrates a protrusion of CDR loops for interacting with the toxin. Zoomed regions show interactions of potential side-chains (ball-and-stick) of V_H_H37 with three spatially juxtaposed areas of the CyaA-RTX/BI-II segment (surface representations colored in cyan, orange and purple) via hydrogen and ionic bonds (dotted lines).

When the CyaA-RTX/BI-II model was docked individually with its specific nanobodies, all nanobodies were found to interact explicitly with several residues in three juxtaposed regions of Linker 1 (Figure 5). For example, V_H_H37 which possesses the highest neutralizing activity among the four obtained nanobodies was revealed to bind the toxin through its CDR-1 and CDR-3 loops of which several polar residues form hydrogen and ionic bonds with mostly charged side-chains (Arg^1101^, Asp^1104^, His^1108^, Asp^1110^, Lys^1113^, Glu^1117^) on the CyaA-RTX/Linker 1 region (see Figure 5). Thus, these results substantiate that the RTX-Linker 1 region (Thr^1105^ to Asn^1132^) could conceivably be a potential neutralizing epitope for these four CyaA-Hly-specific nanobodies as also suggested above by phage-mimotope searching (see Figure 4). Moreover, our present findings are in agreement with recent studies which suggested that the CyaA-RTX subdomain contains immuno-dominant regions capable of eliciting neutralizing antibodies, although epitope data for anti-CyaA antisera used in their studies are not yet described [23]. Further studies, to better understand more critical insights into such toxin-nanobody interactions, directed mutagenesis of these putative interaction sites would be of great interest. Taken together, our present data demonstrate for the first time that CyaA-RTX/Linker 1 could serve as a potential neutralizing epitope of CyaA-protective antigen that would be paving the way for future development of peptide-based pertussis vaccines. Moreover, the toxin-neutralizing nanobodies produced in this study would have a potential for design development and further testing of ready-to-use therapeutic antibodies in passive immunization against such toxin-mediated infection.

## 3. Materials and Methods

### 3.1. Preparation of Purified CyaA-Hly

Recombinant 6× His-tagged CyaA-Hly was expressed and purified as described previously [8]. *E. coli* recombinant cells containing pCyaAC-PF/H_6_ plasmid that encodes the His-tagged CyaA-Hly domain were cultured at 30 °C in Terrific broth supplemented with ampicillin (100 μg/mL) and chloramphenicol (34 μg/mL). Protein expression was induced with isopropyl-β-d-thiogalacto-pyranoside (IPTG) at a final concentration of 0.1 mM, and *E. coli* cells were harvested by centrifugation (6000× *g*, 4 °C, 10 min), re-suspended in 50 mM HEPES buffer (pH 7.4) containing 2 mM CaCl_2_ and 1 mM protease inhibitors (phenylmethylsulfonylfluoride and 1,10-phenanthroline), and subsequently disrupted in a French Pressure Cell (10,000 psi). After centrifugation (13,000× *g*, 4 °C, 15 min), the lysate supernatant was analyzed by SDS-PAGE.

CyaA-Hly was purified from the supernatant by using a metal-chelating affinity column (5-mL HisTrap, GE Healthcare Bio-sciences, Piscataway, NJ, USA). The supernatant (~25 mg) was injected into the column pre-equilibrated with 20 mM imidazole (IMZ) in 50 mM HEPES buffer (pH 7.4) containing 2 mM CaCl_2_. The target protein was stepwise-eluted at a flow rate of 1 mL/min with 75 mM and 250 mM IMZ, respectively. All eluted fractions were analyzed by SDS-PAGE and fractions containing CyaA-Hly were pooled and desalted through a PD10 column (GE Healthcare Bio-sciences, Piscataway, NJ, USA). Protein concentrations were determined by Bradford microassay (Bio-RAD, Hercules, CA, USA).

### 3.2. Selection of CyaA-Hly-Specific VH/V_H_H Nanobodies

To select phage clones that display CyaA-Hly-specific VH/V_H_H nanobodies, a single-round phage bio-panning was performed as described previously [19,20] using 0.1 μM of purified CyaA-Hly as the panning antigen. Toxin antigens in 100 μL carbonate buffer (pH 9.6) were added to individual wells of a microtiter ELISA plate (Costar^®^, Corning, NY, USA) placed in a humid chamber and kept at 37 °C for 1 h and at 4 °C overnight. Each well was then washed with PBS (phosphate-buffered saline, pH 7.4) containing 0.5% Tween-20. A humanized-camel phage display library [20] was added (100 μL containing ~5 × 10^11^ pfu) and kept at 25 °C for 1 h. Log phase-grown HB2151-*E. coli* cells (100 μL) was added to the wells containing the CyaA-Hly-bound phages and kept at room temperature for 30 min to allow phage transduction. Phagemid-transformed bacterial clones were selected on Luria-Bertani (LB) agar plate containing 100 μg/mL ampicillin and 2% glucose. *E. coli* colonies were randomly picked from the overnight incubated plate and then screened for the presence of VH/V_H_H coding sequences (*vhs/v_h_hs*) by colony PCR using phagemid-specific primers: R1 (5'-CCATGATTACGCCAAGCTTTGGAGCC-3') and R2 (5'-CGATCTAAAGTTTTGTCGTCTTTCC-3') [20].

The *vh/v_h_h*-positive clones were grown individually under 0.1 mM IPTG-induction in LB broth. E-tagged-VHs/V_H_Hs in the bacterial lysates, expressed under control of the *lac* promoter in pCANTAB5E vector system, were detected by Western blot analysis using rabbit anti-E tag polyclonal antibodies (Abcam, Cambridgeshire, UK). Alkaline phosphatase (AP)-conjugated goat-anti-rabbit IgG (Southern Biotech, Birmingham, AL, USA) and 5-bromo-4-chloro-3-indolyl phosphate (BCIP)/nitro blue tetrazolium (NBT) substrate (KPL, Gaitherburg, MD, USA) were used for the band revelation.

### 3.3. Binding Assays of CyaA-Hly-Specific VH/V_H_H Nanobodies via Indirect ELISA

Each well of an ELISA plate (Costar^**®**^, Corning, NY, USA) was coated with 0.1 μM of purified CyaA-Hly or antigen control (BSA) in 100 µL of carbonate buffer (pH 9.6). After blocking with 3% BSA in PBS, individual *E. coli* lysates containing VHs/V_H_Hs were added into appropriate wells and the plate was kept at 37 °C for 1 h. For detection of bound VHs/V_H_Hs, the wells were sequentially probed with rabbit anti-E tag antibodies (1:3000 dilution, Abcam, Cambridgeshire, UK) and horseradish peroxidase-conjugated goat anti-rabbit IgG (1:5000 dilution, Southern Biotech, Birmingham, AL, USA). Color was developed with 2,2′-azino-bis(3-ethylbenzothiazoline-6-sulphonic acid) substrate (KPL, Gaitherburg, MD, USA) which has a maximum absorbance at 405 nm. The antigen-coated well added with original HB2151-*E. coli* lysates was used as a negative control and the well filled with PBS was used as blank.

### 3.4. Binding Analysis of CyaA-Hly-Specific VH/V_H_H Nanobodies via Western Blotting

The purified CyaA-Hly toxin was subjected to SDS-PAGE and blotted onto a nitrocellulose membrane (NC) which was then cut into strips. After blocking with 5% skim milk in Tris-buffered saline (TBS, pH 7.4), NC strips were incubated with individual *E. coli* lysates containing VH/V_H_H at 25 °C for 1h. To reveal the protein bands bound with VH/V_H_H, the NC strips were probed sequentially with rabbit anti-E tag antibodies (1:3000 dilution) and AP-conjugated goat anti-rabbit IgG (1:5000 dilution, Southern Biotech, Birmingham, AL, USA). Color was finally developed with BCIP/NBT substrates. The NC strip incubated with original HB2151-*E. coli* lysates was used as a negative control.

### 3.5. Sequence Analysis of CyaA-Hly-Specific VH/V_H_H Nanobodies

Sequences of *vh*/*v_h_h* genes in individual phagemid-transformed *E. coli* clones were verified by DNA sequencing. The resulting DNA sequences of individual CyaA-Hly-specific VH/V_H_H nanobodies were deduced into amino acid sequences of which FRs and CDRs were subsequently predicted via the International ImMunoGeneTics information system [24].

### 3.6. Expression and Purification of VH/V_H_H Nanobodies

For large scale production of CyaA-Hly-specific nanobodies, *vh*/*v_h_h* gene sequence was PCR-amplified using forward primer (5’-TACATATGTGCGGCCCAGCCGGCC-3’) and reverse primer (5’-TCTCGAGACGCGGTTCCAGCGGAT-3’) incorporating *Nde*I and *Xho*I sites on the 5’- and 3’-ends of PCR products, respectively. DNA fragment treated with *Nde*I and *Xho*I was subsequently subcloned into *Nde*I and *Xho*I sites of pET32a(+), an expression vector containing 6× His tag and the strong *T*7/*lac* promoter for high-level expression of recombinant proteins. The resulting plasmids were transformed into *E. coli* cells strain BL21 (DE3). Individual VH/V_H_H nanobodies were over-expressed in *E. coli* as described previously [25].

After cell harvesting, the *E. coli* cells expressing individual nanobodies as inclusions were sonicated in PBS (pH 7.4). Inclusions were collected by centrifugation and then solubilized in denaturing buffer (50 mM Na_2_HPO_4_, 300 mM NaCl, 8 M urea, pH 7.0) at 4 °C for 2 h. Solubilized nanobodies were purified using TALON™ Metal Affinity Resin (Clontech Laboratories, Mountain View, CA, USA) under denaturing conditions of 8 M urea. Refolding of the purified nanobodies was performed as described previously [26]. Specificity of refolded purified nanobodies to the CyaA-Hly toxin was verified by indirect ELISA.

### 3.7. In Vitro Neutralization Assays of the CyaA-Hly Toxin

The ability of CyaA-Hly-specific nanobodies to interfere with binding of CyaA-Hly to erythrocyte membranes was assessed by pre-incubating purified CyaA-Hly (~10 nM) with varied concentrations of toxin-specific VH/V_H_H nanobodies or irrelevant (V_H_H specific to NS3/4A protease of hepatitis C virus [17]) at 25 °C for 1 h. Then, 30 µL of sheep erythrocyte suspension (5 × 10^8^ cells/mL in 150 mM NaCl, 2 mM CaCl_2_, 20 mM Tris-HCl, pH 7.4) was added and the mixture was further incubated at 37 °C for 5 h. Erythrocytes incubated with purified CyaA-Hly for 5 h in the absence of nanobody were used as a negative control. Reaction buffer was used as blank while 0.1% Triton X-100 was used for 100% hemolysis. After centrifugation at 12,000× *g* for 2 min, hemoglobin released from the toxin-induced lysed erythrocytes in the supernatant was measured at OD_540_. Percentage of hemolytic activity of tested toxins with/without VH/V_H_H nanobodies was calculated as described previously [16].

### 3.8. Determination of VH/V_H_H-Specific Phage Peptides

Phage mimotopic peptides that bind to the CyaA-Hly specific VHs/V_H_Hs were determined by a Ph.D-12™ phage display peptide library (New England Biolabs, Ipswich, MA, USA) which contains random 12-residue peptides fused to a coat protein (pIII) of M13 phage as described previously [18]. Each well of a 96-well ELISA plate was coated with VHs/V_H_Hs (1 µg in 100 µL of coating buffer) at 4 °C overnight. Unbound proteins were removed by washing with TBS (pH 7.4) and then each well was blocked with 200 µL of 0.5% BSA in TBS for 1 h and washed once with TBS. The phage-display peptide library (diluted 1:100) that had been subtracted with original BL21 (DE3)-*E. coli* lysate was added to the wells coated with the VHs/V_H_Hs and the plate was kept at 25 °C for 1 h. Unbound phages were removed and the wells were washed with TBS containing 0.5% Tween-20 (TBST). The VH/V_H_H-bound phages were eluted with 0.2 M glycine-HCl (pH 2.2) and the pH was brought up immediately by adding a few drops of 2 M Tris-base solution. The phages from each well were inoculated into 20 mL of log phase-grown ER2738-*E. coli* and incubated at 37 °C for 4 h. The bacterial cells were removed by centrifugation (12,000× *g*, 4 °C, 10 min) and the supernatants containing amplified phage particles were precipitated by adding polyethylene glycol/NaCl and kept at 4 °C overnight. Individual precipitates were re-suspended in 100 µL of TBST and used for the next panning round.

Three rounds of the panning were performed. The eluted phages from the third panning round were used to infect ER2738-*E. coli* cells in agarose-overlaid on LB agar plates containing IPTG and 5-bromo-4-chloro-3-indolyl-β-d-galactopyranoside (X-gal) and incubated at 37 °C overnight. Twenty blue plaques on each plate were picked randomly, inoculated individually into 1 mL of 1:100 diluted log phase-grown ER2738-*E. coli* culture and incubated at 37 °C with shaking for 4 h. DNA of each phage clone was extracted from the culture supernatant via phenol/chloroform method and subsequently sequenced. Peptides displayed by individual phage clones (phage mimotope) were deduced from their DNA sequences. Thereafter, the deduced peptides were classified into mimotope types using Phylogeny Clustal W. The sequence of each mimotope type was multiply aligned with CyaA-Hly sequence in order to locate a region analogous to the phage’s mimotopic peptide, *i.e.*, presumptive VH/V_H_H-binding site on the CyaA-Hly (presumptive epitope).

### 3.9. Homology-Based Modeling of VH/V_H_H Nanobodies and CyaA-RTX Segment

Amino acid sequence (residues 1006–1210) corresponding to Block I-Linker 1-Block II of the CyaA-RTX subdomain was submitted to Raptor server (http://raptorx.uchigo.edu). Incorporation of Ca^2+^ ions was performed by fitting the modeled structure to the template molecule of *Pseudomonas* sp. MIS38 lipase (PDB ID: 2ZJ6). FALC-Loop Modeling server (http://falc-loop.seoklab.org) was used for refinement of loop structure. Finally, the model was subjected to energy minimization using GROMOS96 force field. Structure validation of the final model was performed using programs in NIH SAVES server (http://nihserver.mbi.ucla.edu/SAVES/), including PROCHECK, WHATIF, Verify3D and Ramachandran map. 3D models of CyaA-Hly-specific nanobodies were obtained by employing the similar approaches as described above and their templates are presented in results and discussion section.

### 3.10. Molecular Docking between VH/V_H_H Nanobodies and CyaA-RTX Segment

Protein-protein docking between Block I-Linker 1-Block II of CyaA-Hly and each individual CyaA-Hly-specific VH/V_H_H was performed using ClusPro 2.0 (http://cluspro.bu.edu). Molecular docking was predicted in four separate modes including balance, electrostatic-favored, hydrophobic-favored and Van der Waals, and the ones with the lowest energy scores were selected. Docking models were analyzed by using PyMOL program and interaction profiles of the docked results were analyzed via LigPlot+ [27].

## Supplementary Materials

Supplementary materials are available online at www.mdpi.com//2072-6651/8/4/99/s1. Figure S1. (a) Colony-PCR analysis of phage-transformed *E. coli* clones. 600-bp PCR products exclusively yielded by the *vh*/*v_h_h*-positive clones are indicated. M, GeneRuler^TM^ 1 kb DNA ladder (Thermo Scientific, Waltham, MA, USA). Each lane number corresponds to the clone number of phage-transformed *E. coli*; (b) Western blot analysis of lysate supernatants from the *vh*/*v_h_h*-positive *E. coli* clones using anti-E tag antibodies. E-tagged VH/V_H_H nanobodies expressed in the *E. coli* lysates were revealed as protein bands of ~17–22 kDa. M, pre-stained protein standards. Each lane number is referred to as the clone number of *vh*/*v_h_h*-positive *E. coli*. Figure S2. Expression of CyaA-Hly-specific nanobodies in pET vector system. (a) SDS-PAGE (Coomassie brilliant blue-stained 14% gel) analysis of lysates from *E. coli* expressing CyaA-Hly-specific His-tagged VHs/V_H_Hs under the control of *T7*/*lac* promoter; (b) Western blotting of a probed with anti-His tag antibodies. The expected ~17-kDa protein bands of VH/V_H_H nanobodies are indicated. M, pre-stained protein standards. S and I, lysate supernatants and insoluble pellets after centrifugation, respectively.

## Abbreviations

The following abbreviations are used in this manuscript:

CyaA: adenylate cyclase-hemolysin toxin
CDRs: complementarity determining regions
FRs: immunoglobulin frameworks
Ni^2+^-NTA: nickel-nitrilotriacetic acid
RTX: Repeat-in-Toxin
VH/V_H_H: variable heavy chain domain
