# Supplementary material for: Structural Characterization of Humanized Nanobodies with Neutralizing Activity against the Bordetella pertussis CyaA-Hemolysin: Implications for a Potential Epitope of Toxin-Protective Antigen
Source: Toxins (Basel). 2016 Apr 1;8(4):99. doi: 10.3390/toxins8040099 (PMC4848625; doi:10.3390/toxins8040099)
Supplement: Supplementary file 1 [file toxins-08-00099-s001.pdf]

Supplementary Materials: Structural Characterization of Humanized Nanobodies with Neutralizing Activity against the *Bordetella pertussis* CyaA-Hemolysin: Implications for a Potential Epitope of Toxin-Protective Antigen

Aijaz Ahmad Malik [1,2,3], Chompounoot Imtong [2], Nitat Sookrung [4], Gerd Katzenmeier [2], Wanpen Chaicumpa [3,*] and Chanan Angsuthanasombat [2,5,*]

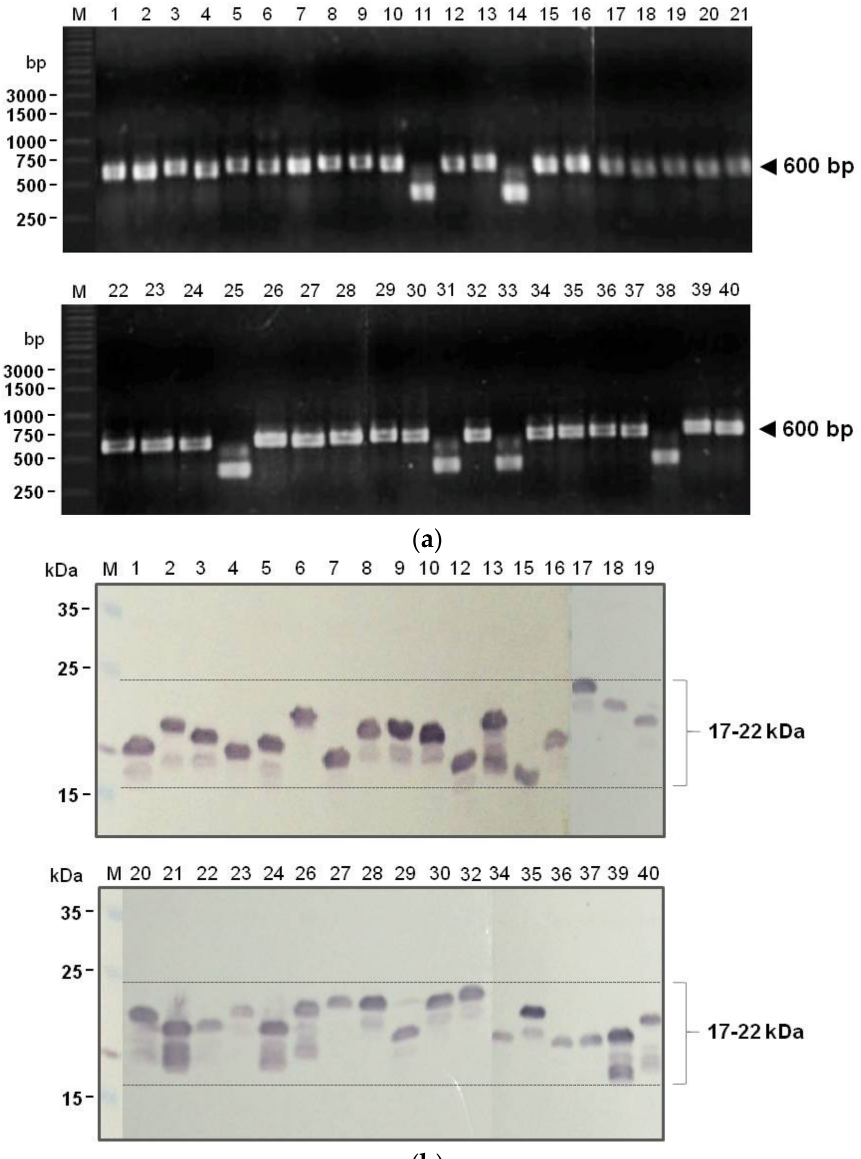

Figure S1. (**a**) Colony-PCR analysis of phage-transformed *E. coli* clones. 600-bp PCR products exclusively yielded by the *vh*/*vhh*-positive clones are indicated. M, GeneRuler™ 1 kb DNA ladder (Thermo Scientific, Waltham, MA, USA). Each lane number corresponds to the clone number of phage-transformed *E. coli*; (**b**) Western blot analysis of lysate supernatants from the *vh*/*vhh*-positive *E. coli* clones using anti-E tag antibodies. E-tagged VH/VHH nanobodies expressed in the *E. coli* lysates were revealed as protein bands of ~17–22 kDa. M, pre-stained protein standards. Each lane number is referred to as the clone number of *vh*/*vhh*-positive *E. coli*.

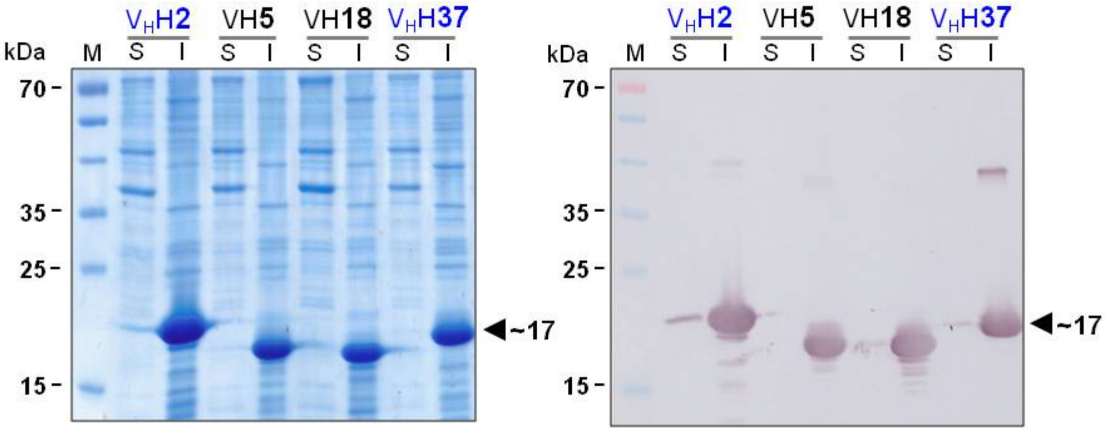

(**a**) (**b**)

Figure S2. Expression of CyaA-Hly-specific nanobodies in pET vector system. (**a**) SDS-PAGE (Coomassie brilliant blue-stained 14% gel) analysis of lysates from *E. coli* expressing CyaA-Hly-specific His-tagged VHs/V_HHs under the control of *T7*/*lac* promoter; (**b**) Western blotting of **a** probed with anti-His tag antibodies. The expected ~17-kDa protein bands of VH/V_HH nanobodies are indicated. M, pre-stained protein standards. S and I, lysate supernatants and insoluble pellets after centrifugation, respectively.
